# Prevalence and Risk Factors of Bovine Fascioliasis in Kelantan, Malaysia: A Cross-Sectional Study

**DOI:** 10.21315/tlsr2021.32.2.1

**Published:** 2021-06-29

**Authors:** Mohamad Ahmad-Najib, Wan Abdul Wahab Wan-Nor-Amilah, Wong Weng Kin, Muhammad Faez Arizam, Noor Jamil Noor-Izani

**Affiliations:** 1 School of Health Sciences, Universiti Sains Malaysia, Health Campus, 16150 Kubang Kerian, Kota Bharu, Kelantan, Malaysia; 2 Faculty of Veterinary Medicine, Universiti Malaysia Kelantan, 16100 Pengkalan Chepa, Kota Bharu, Kelantan, Malaysia

**Keywords:** Bovine, Fascioliasis, Kelantan, Prevalence, Risk Factors, Lembu, Fascioliasis, Kelantan, Prevalens, Faktor Risiko

## Abstract

Bovine fascioliasis is an important zoonotic parasitic disease that causes significant economic losses to the livestock industry. The aim of this study was to determine the prevalence and risk factors of bovine fascioliasis in Kelantan. In this cross-sectional study, a total of 308 stool and blood samples of farmed cattle were collected from December 2017 to June 2018. The stool samples were examined microscopically for the presence of *Fasciola* spp. eggs following a formalin-ether sedimentation process. The blood samples were subjected to a commercial ELISA kit (Bio-X-Diagnostic, Rochefort, Belgium) for the detection of anti-*Fasciola* IgG antibody. The association between coprological findings and risk factors was determined using Pearson’s chi-square (χ^2^). The coproprevalence and seroprevalence of bovine fascioliasis was 14.6% and 37.3%, respectively. There were significant (*P* < 0.05) associations between the risk of infections and the sex, type of feedings, anthelmintic treatment and farm hygiene. Female cattle (OR: 3.104; 95% CI: 1.265, 7.615), feeding by grazing (OR: 4.458; 95% CI: 1.823, 10.90), untreated cattle (OR: 3.833; 95% CI: 1.620, 9.071), non-schedule anthelminthic treatment (OR: 3.927; 95% CI: 1.685, 9.152) and farm that have never been cleaned (OR: 2.829; 95% CI: 1.428, 5.608) showed higher odds of *Fasciola* spp. infection. These findings suggested bovine fascioliasis is a serious veterinary disease in Kelantan. Thus, appropriate control, prevention and monitoring strategies of this parasitic infection are urgently needed to reduce the burden of the disease.

## Abstract (fr)

Fascioliasis pada lembu adalah penyakit parasit zoonotik penting yang menyebabkan kerugian ekonomi yang signifikan pada industri ternakan. Tujuan kajian ini adalah untuk menentukan prevalens dan faktor risiko fascioliasis pada lembu ternak di Kelantan. Dalam kajian keratan rentas ini, sejumlah 308 sampel tinja dan sampel darah daripada lembu ternak telah diambil dari Disember 2017 hingga Jun 2018. Sampel tinja telah diperiksa secara mikroskopik bagi mengesan kehadiran telur *Fasciola* spp. melalui proses sedimentasi formalin-eter. Sampel darah telah disaring menggunakan kit ELISA komersial (Bio-X-diagnostics) untuk pengesanan antibodi anti-*Fasciola* IgG. Hubungan antara penemuan koprologi dan faktor risiko telah ditentukan dengan menggunakan ujian Chi-ganda dua (χ^2^). Koproprevalens dan seroprevalens fascioliasis pada lembu masingmasing adalah 14.6% dan 37.3%. Terdapat perbezaan signifikan (*P* < 0.05) antara risiko jangkitan dan jantina, kaedah pemakanan, penggunaan ubat cacing dan kebersihan ladang. Lembu betina (OR: 3.104; 95% CI: 1.265, 7.615), kaedah pemakanan melalui teknik ragut (OR: 4.458; 95% CI: 1.823, 10.90), lembu yang tidak dirawat (OR: 3.833; 95% CI: 1.620, 9.071), rawatan secara tidak berkala (OR: 3.927; 95% CI: 1.685, 9.152) dan ladang yang tidak pernah dibersih (OR: 2.829; 95% CI: 1.428, 5.608) merupakan antara risiko lebih tinggi untuk dijangkiti *Fasciola* spp.. Penemuan ini mencadangkan bahawa fascioliasis pada lembu adalah penyakit veterinar yang serius di Kelantan. Oleh itu, strategi kawalan, pencegahan dan pengawasan yang sesuai bagi jangkitan parasit ini amat diperlukan untuk mengurangkan beban penyakit ini.

**Highlights**

- Bovine fascioliasis is prevalent in Kelantan, Malaysia.
- The risk of bovine fascioliasis was significantly associated with the sex, type of feedings, anthelmintic treatment and farm hygiene.
- Female cattle, feeding by grazing, untreated cattle, non-schedule anthelminthic treatment, and farms that have never been cleaned showed higher odds of *Fasciola* spp. infection.

## INTRODUCTION

Fascioliasis is an important parasitic disease caused by the trematodes, *Fasciola hepatica* and *Fasciola gigantica*. The trematodes affect a large variety of animals including cattle, sheep, goats and buffaloes through ingestion of infective metacercariae (Hossain *et al*. 2011; Zainalabidin *et al*. 2015; Rahman *et al*. 2017; Mursyidah *et al*. 2017). *Fasciola* species have a complex life cycle which involves snail as an intermediate host where the miracidia develop into thousands of cercariae (Neges & Sahle 2018). Once released from the snails, the cercariae will encyst on aquatic vegetation as metacercariae and taken up by grazing animals. Each metacercaria release an immature fluke which will penetrate the small intestine and migrate through the hepatic parenchyma where it develops into adult and produces eggs.

The disease causes significant economic losses to the livestock industries due to the reduction of body weight, liver condemnation, decrease in milk yield production, increase cost of anthelmintic treatment and reduction of animals’ fertility (Schweizer *et al*. 2005; Khoramian *et al*. 2014; El-Tahawy *et al*. 2017). Fascioliasis has been considered as a worldwide animal health problem as it was reported in both developed and developing countries (Kuerpick *et al*. 2012; Byrne *et al*. 2018; Zewde *et al*. 2019). In Africa, Asia, Oceania and Europe, the occurrence of fascioliasis was highest in cattle compared to other animals such as sheep, goats and buffaloes (Mehmood *et al*. 2017). Fascioliasis in cattle is estimated to cause global economic losses up to million dollars annually. In Europe and Australia, economic losses were from reduction of milk yield and reduced fertility of the livestock (Schweizer *et al*. 2005; Toet *et al*. 2014). In Asia, bovine fascioliasis is spreading widely in many regions including Iran, Iraq, Saudi Arabia, Russia, Thailand, Turkey, China, Vietnam, Nepal, Japan, Korea, Philippines, Pakistan, Bangladesh and Cambodia with prevalence range from 0.71% to 69.2% in cattle and estimated global annual economic losses over 200 million US dollars (Mehmood *et al*. 2017). The respective losses were due to reduced meat production and condemnation of livers (El-Tahawy *et al*. 2017; Zewde *et al*. 2019).

In Malaysia, the prevalence of fascioliasis in cattle has been reported in several states including Johor, Pahang, Perak, Pulau Pinang, Selangor and Terengganu (Saleha 1991; Zainalabidin *et al*. 2015; Khadijah *et al*. 2017; Mursyidah *et al*. 2017; Rita *et al*. 2017; Diyana *et al*. 2019). The prevalence of cattle fascioliasis in Malaysia range from 1% to 95% based on six studies published from 1991 to 2019. The most endemic state in Malaysia is currently Terengganu with a prevalence of 95% (Khadijah *et al*. 2015). The prevalence was based on the surveillance of 40 cattle at two cattle farms located in Kuala Terengganu.

Although bovine fascioliasis has been reported in several states in Malaysia, the epidemiological picture of the disease in Kelantan is still unknown. Kelantan has a conducive tropical climate, with intermittent rain throughout the year and monsoon season from November to January which is a favourable weather for snail population and *Fasciola* spp. life cycle (Mas-Coma *et al*. 2018). Cattle farming are increasingly popular among farmers in Kelantan due to high demand of meat supply. Nevertheless, the possible threat of fascioliasis among farmed cattle in Kelantan is still unknown due to lack of research interest on this neglected tropical disease. Therefore, this study aimed to determine the prevalence and risk factors of *Fasciola* spp. infestation among farmed cattle in Kelantan.

## MATERIALS AND METHODS

### Ethical Approval

The present study protocol was reviewed and approved by the Animal Ethics Committee of Universiti Sains Malaysia (AECUSM). Reference number: USM/IACUC/2017(107)(852).

### Study Area

**Figure 1:**
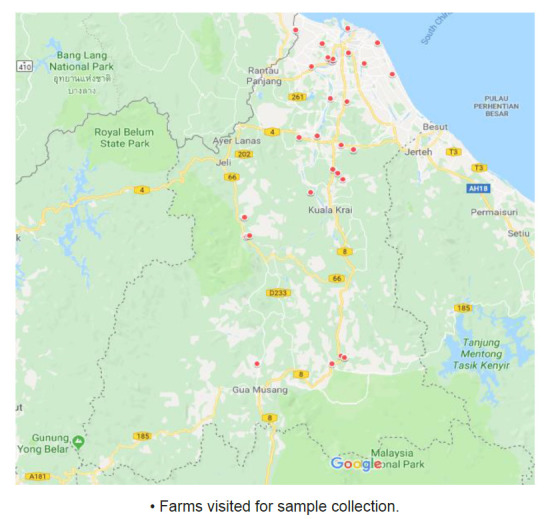
Distribution of copropositive and seropositive cattle in Kelantan. *Source:* Google map

This cross-sectional study was conducted in 10 districts of Kelantan, Malaysia (Fig. 1). Kelantan is one of the 13 states in Malaysia located in the northeast part of Peninsular Malaysia and borders with endemic regions of fascioliasis namely Thailand, Terengganu, Pahang and Perak. A total of 41 farms consisting of six farms in Pasir Mas and Gua Musang, five farms in Kota Bharu, Kuala Krai, Jeli and Machang, three farms in Bachok and Tanah Merah, two farms in Tumpat and one farm in Pasir Puteh were selected for samples collection (Table 1). The selections of farms were made by the officers from the Department of Veterinary Services based on purposive sampling method wherein only farms that have proper animal restrainer and availability of at least 10 cattle in the farm were visited.

### Study Population and Sample Collection

A total of 308 cattle were randomly selected from December 2017 to June 2018. The minimum sample size was based on the single proportion sample size calculation with estimated prevalence of 11.02%, desired margin of error of 3.67%, confidence level of 95% and dropout of 10% (Chakraborty & Prodhan 2015; Masrin *et al*. 2015; Zainalabidin *et al*. 2015). About 30 to 32 cattle of six months of age and above were selected in each district. The fresh stool samples were collected from the rectum of the cattle and transferred into labelled 60 mL stool containers prior to coprological examination. The blood samples were withdrawn from the jugular veins of the cattle into 10 mL plain blood tubes and allowed to clot at room temperature for 2 h followed by centrifugation for 10 min at 1000× g. About 2 mL of serum was aliquoted into a labelled 2 mL microcentrifuge tubes and kept at −20°C.

### Collection of Demographic Data

A set of questionnaires was used to record data on animals’ age, sex, breed, type of farm, source of animals’ drinking water, type of food, frequency of anthelmintic administration and farms’ hygiene. The age of the cattle was determined on the basis of farm’s records. The sex of the cattle was determined by examining the presence of sexual organ. The breed of the cattle, source of animals’ drinking water, type of food, frequency of anthelmintic administration and farms’ hygiene were determined by the consultation with the farms’ owners. The selected cattle farms were grouped into two groups: Dry farms and wet farms, based on the ability of the soil to sustain water.

### Coprological Examination

The stool samples were subjected to formalin-ether sedimentation technique for the detection of *Fasciola* spp. eggs (El-Tahawy *et al*. 2017). The sediment was viewed at 100× and 400× magnification using a light microscope with eyepiece reticle (Zeiss, Germany). Identification of the *Fasciola* spp. egg was made based on the morphology as described in a previous study (Hussein *et al*. 2010). *Fasciola* spp. eggs appeared as golden shiny colour, ellipsoidal, operculated and measured 130 μm–150 μm long by 60 μm–90 μm wide. Interpretation of a positive result for *Fasciola* spp. infestation was based on the presence of *Fasciola* spp. eggs in the stool samples. The microscopic positive slides were verified by two experienced veterinary parasitologists.

### Serological Detection of Anti-*Fasciola* IgG Antibodies

Detection of IgG antibodies against *Fasciola* spp. in serum samples of the cattle was conducted by using a commercial ELISA kit purchased from Belgium, Mono Screen Ab ELISA (Bio-X Diagnostics). The assay was performed according to manufacturer’s instructions (Yildirim *et al*. 2007; Yasar & Burcak 2018). The positivity levels were categorised as no infestation (less than 15%), low infestation (15% to 45%), moderate infestation (45% to 75%) or heavy infestation (greater than 75%). The degree of positivity of each sample was determined by dividing the optical density value with the corresponding positive control and multiplies with 100 to express it as a percentage.

### Statistical Analysis

Data analyses were performed using SPSS version 24.0 (IBM Corporation, Chicago, United States). The prevalence of *Fasciola* spp. infestation in cattle was calculated as percentage value. The associations between epidemiological data and *Fasciola* spp. infestation were determined using Pearson’s chi-square (χ^2^). A statistical association between variables was considered significant if the calculated *P*-value was less than 0.05 with 95% confidence level. A binary logistic regression analysis and 95% confidence intervals (CI) of odds ratio were calculated to quantify the association of the statistically significant variables. A probability level of 5% was used as statistical significance.

## RESULTS

**Figure 2:**
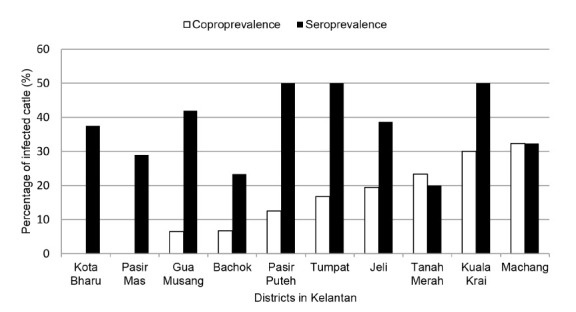
Distribution of anti-*Fasciola* IgG antibody titre among cattle.

The coprological examination of the cattle stool samples showed 14.6% (45/308) positive *Fasciola* spp. eggs. Positive coproscopy results were found in eight out of the ten Kelantan districts, namely Gua Musang, Tumpat, Bachok, Pasir Puteh, Tanah Merah, Kuala Krai, Jeli and Machang. The highest coproprevalence of fascioliasis among cattle was recorded in Machang with prevalence of 32.3% (Fig. 2).

**Figure 3:**
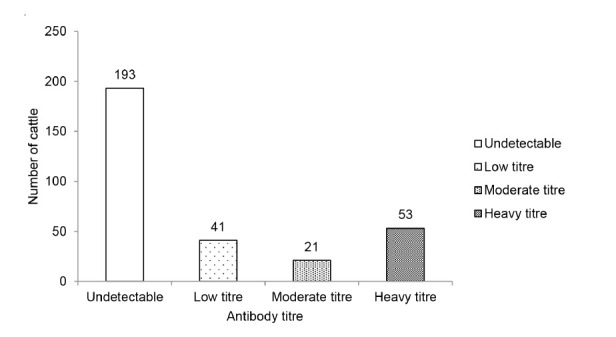
Distribution of anti-*Fasciola* IgG antibody titre among cattle.

In serological study, 37.3% (115/308) of the cattle serum samples were positive for anti-*Fasciola* IgG antibody. Seropositive cases were detected among cattle from all 10 districts in Kelantan. The highest numbers of seropositive cases were recorded in Pasir Puteh, Kuala Krai and Tumpat, with prevalence up to 50% in all three districts. Tanah Merah recorded the lowest prevalence of 20% (6/30). Of the 115 seropositive cases, 36% had a low antibody titre, 18% had a moderate antibody titre and 46% had a high antibody titre (Fig. 3). Of the 41 farms visited, 32 farms (78%) were positive for anti-*Fasciola* antibody. Out of 115 seropositive cattle, eggs were only found in 37 (32%) cattle with 18 (49%) cattle had a high titre of the antibody, 6 (16%) cattle had a moderate titre of antibody and 13 (35%) cattle had a low titre of antibody (Table 2).

The results of the association analysis of epidemiological data with the prevalence of *Fasciola* spp. infestation are presented in Table 3. There was a significant association between *Fasciola* spp. infestation and sex (*P* = 0.01). The female cattle have 3.104 times odds of *Fasciola* spp. infestation as compared to the male cattle. Higher prevalence of *Fasciola* spp. infestation was observed among those cattle that were allowed for grazing. Cattle fed via grazing was at 4.458 times odds to have *Fasciola* spp. infestation (*P* = 0.001) as compared to those fed on silage. With regard to the anthelmintic practice, the prevalence of *Fasciola* spp. infestation was higher among those cattle that have never received anthelmintic and those cattle that were only given anthelmintic when suspected of being infected with worms (*P* = 0.004). Cattle that have never been administered with anthelmintic drugs are at 3.833 times odds of *Fasciola* spp. infestation, as compared to cattle given anthelmintic drugs every six months. Besides, cattle that were only given anthelmintic drugs when suspected of being infected with worms demonstrated a higher risk for *Fasciola* spp. infestation with 3.927 times odds as compared to cattle given anthelmintic drugs every six months. Regarding farm cleaning, cattle raised in farms that have never been cleaned demonstrated higher prevalence of *Fasciola* spp. infestation with 2.829 times odds than those raised in farms that are cleaned daily (*P* = 0.003).

## DISCUSSION

Fascioliasis is a serious threat to the livestock industry due to significant economic losses that attribute to liver condemnation and growth retardation of infected cattle (Kozlowska-Loj & Loj-Maczulska 2013; El-Tahawy *et al*. 2017; Arbabi *et al*. 2018). The present study showed the prevalence of *Fasciola* spp. infestation in cattle was 14.6% based on coprological examination and 37.3% based on serological detection of anti-*Fasciola* IgG antibody. The coproprevalence of *Fasciola* spp. infestation among cattle in the present study is lower than those in the previous studies in Terengganu (Khadijah *et al*. 2015; 2017). The higher prevalence in the previous studies in attributed mainly to the smaller samples size which increase margin of error in the studies (Naing *et al*. 2006). The coproprevalence of *Fasciola* spp. infestation in this study was similar to studies found in endemic countries such as Egypt (28.6%) and Vietnam (23.4%) (Hussein & Khalifa 2010; Nguyen *et al*. 2017). This indicates that cattle fascioliasis is a serious problem in Kelantan. Therefore, prevention and control measures need to be taken immediately to overcome the burden of the infestation.

The prevalence detected by the serological approach was higher than that detected by the traditional coprological examination. The difference between the coproprevalence and seroprevalence of the infestation was similarly reported in a study in Turkey which showed higher prevalence based on serological approaches compared to the coprological method (Yildirim *et al*. 2007). The explanation for this difference is that anti-*Fasciola* spp. antibodies can be detected much earlier than the detection of *Fasciola* spp. egg which is only present after 12 weeks post infestation (Mohammed *et al*. 2018). Besides, antibodies also remain in infected cattle for up to six months post infestation (Castro *et al*. 2000). Therefore, cattle that were previously exposed and recovered from the infestation will also show presence of anti-*Fasciola* antibody.

An interesting result found in the present study was that 18% of the cattle positive for coprological examination were found negative by serological detection of anti-*Fasciola* spp. IgG antibody. These findings may reflect a low immune response to the antigenic stimulus from the migrating or mature flukes in the cattle which result in undetectable antibody (Afshan *et al*. 2013). Another explanation was a false positive coproscopic result due to ingestion of pasture contaminated with *Fasciola* spp. eggs (John *et al*. 2019). This study also found that antibody titre does not correlate with the presence of eggs in the stool. All ranges of antibody titres were observed among copropositive cattle. This observation was in agreement with the previous study in Turkey which demonstrated various titres of antibody among copropositive cattle (Yildirim *et al*. 2007).

The present study showed no significant association between *Fasciola* spp. infestation and cattle age. This finding is in agreement with the previous studies in Vietnam and Nigeria (Nguyen *et al*. 2017; Shinggu *et al*. 2019). However, several studies reported different results whereby higher prevalence was seen in cattle of above five years old compared to cattle aged below five years old (Rita *et al*. 2017). The plausible reason for the differences is the feeding techniques practiced by farmers. The cattle aged above five years old was more likely to be infested due to the cattle aged above five years old were let to graze freely around the farm making them more exposed to the causative agent of the infestation (Rita *et al*. 2017).

With regards to cattle sex, the present study showed female cattle have higher odds of association with *Fasciola* spp. infestation as compared to male cattle. This observation is in agreement with the previous studies in Terengganu which reported higher prevalence of *Fasciola* spp. infestation in females than males (Rita *et al*. 2017; Mohammed *et al*. 2018). This might be due to most female cattle were kept for milking the young which can be considered as a stressful physiological factor that may affect their immunity against the infestation. In contrary, a study in Egypt found a significantly higher prevalence of *Fasciola* spp. infestation in males than females (El-Tahawy *et al*. 2017). The observation may probably be due to the practice of keeping females under healthier and nourishing conditions compared to males which are kept free to graze on the fields (Khan & Maqbool 2012).

In the present study, grazing was found to have a statistically significant association with *Fasciola* spp. infestation in which it showed more than four times the odds compared to silage. The reason for that is due to feeding silage helps in removing metacercaria attached to the grass before it is given to their livestock (John *et al*. 2019). This finding suggests that feeding silage is an effective strategy for the prevention and control of the infestation. The present study also showed that there was no significant association between the source of water and *Fasciola* spp. infestation. On the contrary, several studies reported that river as the main water source for the transmission of fascioliasis (Mas-Coma *et al*. 2018). This outcome revealed that the infestation sources vary according to geographical areas, depending on suitable environmental condition for the survival of intermediate hosts and infective stage metacercariae (Neges & Sahle 2018).

Undoubtedly, the animals which did not receive anthelmintic (albendazole, fenbendazole and macrocyclic lactone) treatment for fascioliasis are of higher risk of fascioliasis than the one with regular treatment. A higher prevalence of fascioliasis was observed among cattle without anthelmintic treatment (Nguyen *et al*. 2017). *Fasciola* spp. requires a host to complete its life cycle and grows its population in an area. Therefore, the regular administration of anthelmintic is important in ensuring effective control to prevent the outbreak of infestation. However, over-reliance on anthelmintic could lead to a serious problem which causes the development of anthelmintic resistance (Kelley *et al*. 2016). Understanding of confounding factors such as correct dosing, treatment duration and regular surveillance is now critical for effective management of the drug resistance.

This study also found a significant association between coprological findings and frequencies of farm cleaning. In this study, a farm cleaning refers to the disposal of animal waste from the farm areas. The prevalence of *Fasciola* spp. infestation among cattle in the daily-cleaned farms was lower than cattle in the farms that have never been cleaned. The practice of cleaning prevents the healthy cattle from being exposed to *Fasciola* eggs shed by the infested cattle, which might increase the risk of disease transmission especially in the grazing areas (Gupta 2014). Therefore, hygiene interventions on farms’ area must be implemented to prevent a sustained transmission of *Fasciola* spp.

## CONCLUSION

In conclusion, the present study unravelled the current status of *Fasciola* spp. infestation among farmed cattle in Kelantan. The epidemiological investigation of risk factors revealed a significant association between *Fasciola* spp. infestation with the sex, feeding techniques, frequency of anthelmintic treatment and farms’ hygiene. Thus, the outcome of this study will contribute to helping cattle farmers and veterinary department in implementing effective strategies to control the risks of fascioliasis among farmed cattle in Kelantan.

## Figures and Tables

**Table 1:** Number of farms and cattle per district.

Districts	Number of farms	Number of cattle
Kota Bharu	5	32
Pasir Mas	5	31
Gua Musang	6	31
Kuala Krai	6	30
Jeli	5	31
Machang	5	31
Bachok	3	30
Tanah Merah	3	30
Tumpat	2	30
Pasir Puteh	1	32

Total	41	308

**Table 2:** Number of *Fasciola* spp. infestation cases by serology and coprological examination.

	Copropositive	Copronegative
Undetectable anti-*Fasciola* IgG antibody	8	185
Low titre of anti-*Fasciola* IgG antibody	18	23
Moderate titre of anti-*Fasciola* IgG antibody	6	15
High titre of anti-*Fasciola* IgG antibody	13	40

Total	45	263

**Table 3:** Risk factors associated with *Fasciola* spp. infestation among cattle in Kelantan.

Attribute	*N* (%)	*Fasciola* spp. infestation		Odds ratio	CI (95%)	*P-* value

		Positive *N* (%)	Negative *N* (%)			
Age (Months)						0.210
0–24	186 (60)	22 (12)	164 (88)			
25–36	75 (25)	15 (20)	60 (80)			
>36	47 (15)	8 (17)	39 (83)			
Sex						0.010*
Male (Reference)	91 (30)	6 (7)	85 (93)			
Female	217 (70)	39 (18)	178 (82)	3.104	1.265, 7.615	
Breed						
Kedah-Kelantan	213 (69)	36 (17)	177 (83)			0.231
Charolaise	76 (25)	7 (9)	69 (91)			
Others (Limousine and Brahman)	19 (6)	2 (11)	17 (89)			
Type of farm						0.286
Dry	193 (63)	25 (13)	168 (87)			
Wet	115 (37)	20 (17)	95 (83)			
Source of water						0.187
River	66 (21)	13 (20)	53 (80)			
Non-River	242 (79)	32 (13)	210 (87)			
Type of feed						0.001*
Silage (Reference)	113 (47)	6 (5)	107 (95)			
Grazing	195 (63)	39 (20)	156 (80)	4.458	1.823, 10.900	
Anthelmintic administration						0.004*
Every six months (Reference)	150 (49)	12 (8)	138 (92)			
Never	52 (17)	13 (25)	39 (75)	3.833	1.620, 9.071	
Only when suspect an infestation	55 (18)	14 (26)	41 (74)	3.927	1.685, 9.152	
Annually	32 (10)	4 (13)	28 (87)			
Every three months	19 (6)	2 (10)	17 (90)			
Farm cleaning						0.003*
Daily (Reference)	150 (49)	14 (9)	136 (91)			
Weekly	25 (8)	1 (4)	24 (96)			
Never	133 (43)	30 (23)	103 (77)	2.829	1.428, 5.608	

*Note*: Pearson *χ*^2^ analysis,

* statistically significant if *P*-value <0.05.
